# Molecular Phylogenetic Analysis of Non-Sexually Transmitted Strains of *Haemophilus ducreyi*

**DOI:** 10.1371/journal.pone.0118613

**Published:** 2015-03-16

**Authors:** Jordan R. Gaston, Sally A. Roberts, Tricia L. Humphreys

**Affiliations:** 1 Department of Biology, Allegheny College, Meadville, Pennsylvania, United States of America; 2 Department of Microbiology, Auckland District Health Board, Auckland, New Zealand; University of Ottawa, CANADA

## Abstract

*Haemophilus ducreyi*, the etiologic agent of chancroid, has been previously reported to show genetic variance in several key virulence factors, placing strains of the bacterium into two genetically distinct classes. Recent studies done in yaws-endemic areas of the South Pacific have shown that *H*. *ducreyi* is also a major cause of cutaneous limb ulcers (CLU) that are not sexually transmitted. To genetically assess CLU strains relative to the previously described class I, class II phylogenetic hierarchy, we examined nucleotide sequence diversity at 11 *H*. *ducreyi* loci, including virulence and housekeeping genes, which encompass approximately 1% of the *H*. *ducreyi* genome. Sequences for all 11 loci indicated that strains collected from leg ulcers exhibit DNA sequences homologous to class I strains of *H*. *ducreyi*. However, sequences for 3 loci, including a hemoglobin receptor (*hgbA*), serum resistance protein (*dsrA*), and a collagen adhesin *(ncaA)* contained informative amounts of variation. Phylogenetic analyses suggest that these non-sexually transmitted strains of *H*. *ducreyi* comprise a sub-clonal population within class I strains of *H*. *ducreyi*. Molecular dating suggests that CLU strains are the most recently developed, having diverged approximately 0.355 million years ago, fourteen times more recently than the class I/class II divergence. The CLU strains' divergence falls after the divergence of humans from chimpanzees, making it the first known *H*. *ducreyi* divergence event directly influenced by the selective pressures accompanying human hosts.

## Introduction

*Haemophilus ducreyi* is a Gram-negative coccobacillus in the polyphyletic family *Pasteurellaceae*, and is the etiologic agent of the sexually transmitted infection chancroid, a genital ulcer disease [1]. An obligate human pathogen, H. ducreyi has no known environmental reservoir outside of its host. Like other ulcerative sexually transmitted infections, chancroid facilitates both the transmission and acquisition of HIV-1, making the limitation of chancroidal infection paramount for HIV control efforts [2–5]. Chancroid ulcers are able to facilitate transmission of HIV-1, making chancroid a concern to HIV prevention efforts due to is presence among commercial sex worker populations ([2–5]

*H*. *ducreyi* has historically only been known to cause genital ulcer infections as chancroid is transmitted via micro-abrasions developed during sexual intercourse [1]. However, in 1989, an ulcer caused by *H*. *ducreyi* was reported on the left foot of a 22-year-old male reported to have returned to Denmark from the Fiji Islands who had no history or sign of primary genital infection [6]. No additional reports of non-genital *H*. *ducreyi* infection were reported until 2007, when *H*. *ducreyi* was confirmed to be the etiologic agent of cutaneous limb ulcers (CLU) present in three children ranging between 6–9 years of age via phenotypic testing on cultures and by 16S rDNA sequencing [7]. These cases also occurred after travel to the South Pacific, as each child had recently visited Samoa. In 2007, another extra-genital leg ulceration due to *H*. *ducreyi* was reported in a 5-year-old Sudanese refugee recently placed in Canada. Symptoms were similar to those seen in previous *H*. *ducreyi* leg ulcerations, and the infection was resolved after treatment with oral azithromycin [8]. This case was the first reported outside of the South Pacific, indicating *H*. *ducreyi* associated leg ulceration may be more widespread than previously suspected. Chronic limb ulcers caused by *H*. *ducreyi* are not limited to children [9]. [10].

Recent studies conducted in the South Pacific Island Countries and Territories (PICT) have shown that *H*. *ducreyi* is the etiologic agent of non-sexually transmitted skin ulcers which clinically resemble yaws, an infection by *Treponema pallidum* subspecies *pertenue*. A prospective cohort examination conducted in the yaws-endemic area Papua New Guinea revealed *H*. *ducreyi* is the cause of 3.2 cases per 100 persons, with *H*. *ducreyi* DNA detected in 60% of ulcers and *T*. *pallidum* DNA detected in 34% [11]. These data suggest *H*. *ducreyi* is a major cause of leg ulcers. While often characterized as fastidious, *H*. *ducreyi* is first and foremost a skin pathogen, which has been demonstrated by experimentally established infections in the upper arms of human volunteers [12]. Thus, a non-genital infection would not be entirely unprecedented. Similar findings were also reported in a yaws survey conducted in the Solomon Islands [13].

These new infection patterns prompt a re-examination of the population structure of *H*. *ducreyi*. While many *Haemophilus* species are inherently competent, *H*. *ducreyi* is considered non-transformable due to mutations within its competence genes, *comA*, *comB*, and *comM*, which contain an internal stop codon [14]. Thus, rates of recombination and gene transfer are likely lower than other species, generating stable clones among *H*. *ducreyi* strains [14,15]. Previous attempts at examining genetic diversity within the species *H*. *ducreyi* using PCR and sequencing analysis have shown a large amount of genetic variation between a few key virulence genes within the genome of *H*. *ducreyi*, and strains of *H*. *ducreyi* fall into the recognized subcategories class I and class II [16–18]. Here we re-examine the relationships among *H*. *ducreyi* strains using a Multi Locus Sequence Analysis (MLSA) of 11 genes, including virulence factors and housekeeping genes, to understand the position of strains cultured from cutaneous limb ulcers within the class hierarchy [19].

## Methods

### Strains and Culture Conditions

*H*. *ducreyi* strains were cultured on Columbia Agar supplemented with 5% hemoglobin and 1% IsoVitaleX (Table 1). Strains were revived from frozen stocks and incubated at 35°C with 5% CO_2_ for 24–48 hours. Strains were sub-cultured no more than once prior to PCR.

**Table 1:** Strains of *H. ducreyi* used in this study.

**Strain**	**Class**	**Site of Isolation (year)**	**Reference**
NZS1	CLU^1^	Samoa (2006)	[7]
NZS2	CLU	Samoa (2006)	[7]
NZS3	CLU	Samoa (2006)	[7]
NZS4	CLU	Samoa (2007)	This study
SSMC57	I	Bangladesh (N/A^2^)	[18]
82–029362	I	California, USA (1982)	[31]
HMC56	I	Dominican Republic (1995)	[32]
HMC60	I	Florida, USA (1989)	[32]
HD188	I	Kenya (1982)	[31]
HMC46	I	Kenya (1995)	[32]
6644	I	Massachusetts, USA (1989)	[31]
C111	I	Nairobi, Kenya (N/A)	[18]
85–023233	I	New York, USA (1985)	[31]
HD183	I	Singapore (1982)	[31]
35000HP	I	Winnipeg, Canada (1972)	[33]
DMC111	II	Bangladesh (N/A)	[18]
DMC64	II	Bangladesh (N/A)	[18]
SSMC71	II	Bangladesh (N/A)	[18]
CIP542	II	Hanoi, Vietnam (1954)	[18]
33921	II	Nairobi, Kenya (N/A)	[16]
HMC112	II	Unknown (1984)	[18]

^1^ *H*. *ducreyi* strains isolated from a cutaneous limb ulcer

^2^ N/A, year of isolation unknown

### PCR, Gel Electrophoresis, and Sequencing

Genomic DNA was obtained from each strain using GeneReleaser according to the manufacturer’s specifications (BioVentures, Murfreesboro, TN). Sequences of all loci (*16s rDNA*, *cpxR*, *wecA*, *recA*, *dsrA*, *ncaA*, *ompA2*, *lspA2*, *fgbA*, *hgbA*, *dltA)* were obtained from fragments amplified via PCR using the primers listed (Table 2). PCR products were examined for quality and contamination via gel electrophoresis. Gels were stained with SYBR Safe (Life Technologies, Grand Island, NY) and quantified using ImageJ to determine relative concentration of DNA in PCR products prior to sequencing. Monodirectional sequencing was conducted by Eurofins MWG|Operon and Yale University DNA Analysis Facility using the forward primers employed in PCR to amplify loci selected for analysis (Table 2). All sequences for CLU strains were obtained as part of this study. All *cpxR* sequences were obtained as part of this study, as was the *fgbA* sequence for strain 82–029362. All other sequences were obtained from GenBank. See S1 Table for a list of accession numbers for all genes used in this study.

**Table 2:** List of oligonucleotides used in this study.

**Locus**	**Forward**	**Reverse**	**Reference**
*fgbA*	5′ CCTCAAGTTGAAGAAATGAAACAAACGG 3′	5′ CGAATTCGTATTTGGTAATAAATGACCGC 3′	[34]
*wecA*	5′ CCGGAATCACCCATAAACAC 3′	5′ CCGGAATCACCCATAAACAC 3′	[17]
*recA*	5′ CATTATGGCAGCGGATAAAAA 3′	5′ TCCTCAAACGCTTCATCAAA 3′	[17]
*hgbA*	5′ AGCGATTACTCTCTGTATTTTGGGG 3′	5′ GCAGATATTGCTGCATCATCGGAG 3	[18]
*lspA2*	5′ AAGTTTCAGCAAGAGCGGC 3′	5′ TATTGGCTGCAAGCTCTG 3′	[35]
*ncaA*	5′ GGTTGATTATGTCGAATAATTTG 3′	5′ CTAAGCGCGTAAAAATTCGATG 3′	[18]
*dsrA*	5′ GACAGCATTCAGTGAATAATGGC 3′	5′ AATGAAGTCCGCACCTTTAACGGC 3′	[18]
*ompA2*	5′ GAGGTACCGCGCCACAAGCGGATACTTTTTAT 3′	5′ GCTTAAGCGTGGTTTATCTCTTACATTCGCTACA 3′	[26]
*dltA*	5′ CGCTTGTACAAGCGGGC 3′	5′ CAGCTTACAAAATGATGGGC 3′	[36]
*16s rDNA*	5′ CAAGTCGAACGGTAGCACGAAG 3′	5′ TTCTGTGACTAACGTCAATCAATTTTG 3′	This Study
*cpxR*	5′ TGAAGATCCAAGAGGCAATG 3′	5′ AGGGATTCGTTCAATCGACA 3′	This study

### Computational Analyses

DNA sequences were edited for base miscalls and trimmed of low-quality bases in Sequencher 5.2 (Genecodes, Ann Arbor, MI). Due to the length of the *hgbA* locus, internal primers were used to primer walk sequencing as previously described [18]. *hgbA* sequences were then assembled into consensus sequences representing the entire region of interest. Alignments of each loci were also initially performed in Sequencher 5.2 and all sequences were further trimmed so that each amplicon was of equal length. Alignments were exported in FASTA format, and imported into MEGA 6.0 for phylogenetic analysis (Neighbor-Joining, Minimum Evolution, Maximum Likelihod, and Maximum Parismony) [20]. A bootstrap consensus neighbor-joining tree was generated for loci containing informative variation using the Tamura-3 Parameter statistical method and tested for significance using 1,000 rounds of bootstrap replication. Tests for selection and nucleotide diversity were carried out in MEGA 6.0 as well using Tajima’s Test of Neutrality to calculate Tajima’s D statistic. Nucleotide sequences for *dsrA* and *ncaA* were translated to amino acids in MEGA 6.0 and examined for variation. The relative clade divergence date for loci not under positive selection was calculated by dividing the number of single nucleotide polymorphisms (SNPs) by the total number of base pairs then dividing the quotient by the evolutionary rate (1% per million years) of the closest relative possible [21,22].

## Results

**Fig 1:**
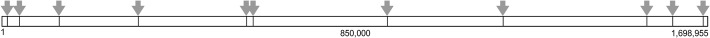
Distribution of sequenced loci compared to the 35000HP reference genome. A graphic depiction of the distribution of loci chosen for MLSA analysis. Sites are aligned to the complete 35000HP reference genome (horizonal bar). All sites chosen for analysis were unlinked, and represent a genome wide distribution of variation. Arrows represent loci chosen for analysis. Numbers represent position in base pairs relative to the reference genome.

In this study, a modified MLSA protocol was used to characterize *H*. *ducreyi* isolates from non-genital infections (Table 3). The sequences from multiple strains of class I and class II *H*. *ducreyi* were previously published for 7 of the loci examined in this study: *wecA*, *recA*, *fgbA*, *hgbA*, *ncaA*, *dsrA*, and *lspA2* [17]. To expand this data set, we determined the DNA sequences for all of the above loci from the genomes of four leg ulcer isolates. Four genes previously used to distinguish class I and II (*pal*, *murC*, *mtrC*, *sapA*) had very limited variation even between class I and II [17], and were therefore excluded from this analysis. In place of these four genes, we examined four additional loci, *cpxR*, *ompA2*, *dltA*, and *16s rDNA*, to better distinguish the CLU (cutaneous limb ulcer) strains from class I and class II *H*. *ducreyi*. These additional loci also increased the distribution of loci throughout the genome. Thus, the total data set includes 11 unlinked loci with genome wide distribution in class I, class II, and CLU strains (Fig. 1). This number exceeds the standard conventional multilocus sequence analysis that requires the sequencing and comparison of 7 unlinked loci [19], and is sufficient to establish *H*. *ducreyi* CLU strains' position among the previously described class hierarchy of strains [17].

**Table 3:** Cutaneous limb ulcer patient information.

**Strain**	**Year**	**Gender**	**Age (yr)**	**Clinical Outcome**
NZS1	2006	F	11	ulcer healed following antimicrobial treatment
NZS2	2006	F	5	ulcer healed following antimicrobial treatment
NZS3	2006	F	6	ulcer healed following antimicrobial treatment
NZS4	2007	F	15	ulcer healed following antimicrobial treatment

**Fig 2:**
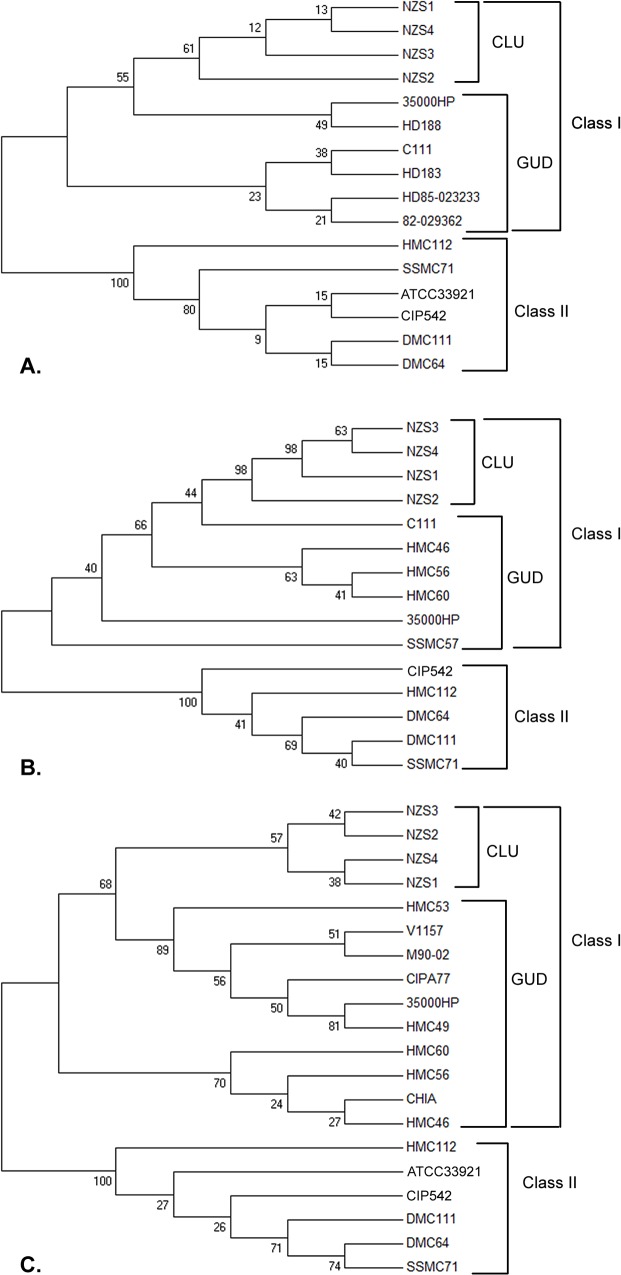
Evolutionary relationships of taxa using the loci (A) *ncaA*, (B) *hgbA*, and (C) *dsrA*. The evolutionary history was inferred with the Neighbor-Joining method using MEGA6. The bootstrap consensus tree above were inferred from 1,000 replicates. Bootstrap values are located on top of the nodes.

While neighbor-joining trees were used as final phylogenies, other methods of phylogeny reconstruction (Maximum Likelihood, Maximum Parsimony, Minimum Evolution) showed similar topology and positioning of CLU strains among class I *H*. *ducreyi*, with minor differences in the bootstrap values and strain positioning within class clades. CLU strains of *H*. *ducreyi* form a statistically supported subclade within the class I clade of *H*. *ducreyi*. Thus, they are the only series of *H*. *ducreyi* strains which form a subclade based on their geographic location (Table 1, Fig. 2). *cpxR*, *ompA2*, *lspA2*, *16s rDNA*, *dltA*, *fgbA*, *recA*, and *wecA* were excluded from phylogenetic reconstruction due to a lack of informative variation needed to produce high fidelity tree branches.

Concatenation of the 11 genes provided a total of 11,398 sites, representing ∼0.67% of the *H*. *ducreyi* genome when compared to the size of the published 35000HP genome (NC_002940.2). Variation specific to CLU strains was present within several of the loci surveyed, with 74 sites differentiating CLU strains from class I. Most of the genetic variation was concentrated in 3 of the 11 genes surveyed (*ncaA*, *dsrA*, *hgbA)*, all of which code for proteins involved in bacterial virulence in humans. Though variation was present, the majority of nucleotides were conserved between class I and CLU *H*. *ducreyi*. *wecA*, *cpxR*, *fgbA*, *16S rDNA*, and *dltA* CLU sequences contained no variability when compared to class I sequences. *recA* contained 1 polymorphic site, *ompA2* had 2 (2.7%), and *lspA2* contained 5 variable sites (6.7%) when compared to class I sequences. Of the total variablility specific to the CLU strains, *ncaA* contained 40 variant sites (54.1%), *dsrA* contained 18 variant sites (24.3%), and *hgbA* contained 7 variant sites (9.5%). CLU strains were not monotypic at these loci as differential haplotypes are present within each of the loci, comprised of either one or two CLU strain-specific single nucleotide polymorphisms. When compared to class II strains of *H*. *ducreyi*, CLU strain sequences contained no similar SNPs to those characteristic of class II.

With the exception of CLU *dsrA (D = 2*.*773)* and *hgbA* (D = 2.156), which were found to be under positive selection when grouped with only class I strains, all loci studied were under neutral or purifying selection (Table 4). Due to the high amounts of variation between class I and class II *H*. *ducreyi* within the *dsrA* and *hgbA* loci, only class I sequences were chosen for statistical comparison to CLU *H*. *ducreyi* during selection analyses to eliminate bias from class divergence (Table 4). Thus, the Tajima’s D statistic gives a comparative depiction of selection between CLU and class I strains. While genes under positive selection are normally not suitable for MLSA analysis [19], the gene loci under positive selection were included for phylogenetic reconstruction as the CLU sequences contained numerous informative sites while retaining a high level of conservation when compared to class I *H*. *ducreyi*. CLU strains contained less nucleotide diversity when compared to class I than when class I is compared to class II *H*. *ducreyi*. In between group comparison, CLU and class I strains had an average nucleotide diversity of 0.00220, while the average nucleotide diversity between class I and class II was 0.0479.

**Table 4:** Nucleotide diversity and selection relative to location and function.

**Locus**	**Position** ^1^	**Length** ^2^	**Tajima's D** ^3^	**Class I vs. Class II (π)** ^4^	**Class I vs. CLU (π')**	**Location; Function**
*16s rDNA*	10643–12179	396	0.85056	0	0.00133	Cytoplasm; ribosomal subunit
*cpxR*	1204379–1205101	598	−0.27919	0.00447	0.00114	Cytoplasm; response regulator
*dltA*	586988–597714	697	1.70281	0.00719	0	Outer membrane; Lectin
*dsrA*	603307–604195	1229	2.77342	0.16910	0.01420	Outer membrane; serum resistance
*fgbA*	136574–137285	648	0.29421	0.00795	0	Outer membrane; fibrinogen binding
*hgbA*	1686700–1689521	2860	2.15658	0.01859	0.00049	Outer membrane; hemoglobin receptor
*lspA2*	925194–925452	461	−1.40008	0.10658	0.00166	Outer membrane; antiphagocytic protein
*ncaA*	1614393–1614596	2138	−0.78926	0.09943	0.00140	Outer membrane; collagen adhesin
*ompA2*	41761–42984	1000	1.30267	0	0.00040	Outer membrane; membrane stability
*recA*	325798–326862	975	−0.81649	0.00877	0.00055	Periplasm; recombination promotion
*wecA*	1551061–1551456	396	0.33694	0.00456	0.00091	Cytoplasm; synththesis of common antigen
Weighted Average				0.0479	0.00220	

^1^ Position in base pairs relative to the *H*. *ducreyi* 35000HP genome (NC_002940.2).

^2^ PCR amplicon alignment length in base pairs, including indels.

^3^ Tajima's D statistic of selection; positive values indicate positive selection, and those greater than 2 are significant.

^4^ Tajima's pi statistic; indicates the relative nucleotide diversity at any given locus.

When comparing the sequence of amino acids in each of the loci used for phylogenetic reconstruction, HgbA_CLU_’s amino acid sequence was completely conserved when compared to class I. However,DsrA_CLU_ and NcaA_CLU_ contained unique variation within their amino acid sequences (Table 4). NcaA contained 1 amino acid substitution, while DsrA_CLU_ contained 13 amino acid shifts within the passenger domain of the protein, which is involved in fibrinogen binding [23]. DsrA_CLU_ strains contained a dimer of the *NTHNINK* motif in the serum resistance domain similar to that of class I strains other than M90-02 and V1157, which contain a trimer, and 35000HP, which contains a monomer (Table 5).

**Table 5:** Characterization of nonsynonymous amino acid changes in CLU strains relative to 35000HP (I).

**Locus**	**Location**	**Change**	**Significance**
*ncaA*	Not Characterized	Lys47Glu	Charge Inversion
*dsrA*	Passenger Domain	Gly51Asp	Nonpolar to Polar
	Passenger Domain	Val57Ala	NS^1^
	Passenger Domain	Glu59Lys	Charge Inversion
	Passenger Domain	Ala72Val	NS
	Passenger Domain	Glu76Lys	Charge Inversion
	Passenger Domain	Ala92Val	NS
	Passenger Domain	Gly94Pro	Amino to Imino
	Passenger Domain	Val95Pro	NS
	Passenger Domain	Ser96Pro	Amino to Imino
	Passenger Domain	Pro99Ser	Imino to Amino
	Passenger Domain	Lys136Asn	Positive to Neutral
	Passenger Domain	Tyr155His	Hydrophobic to Hydrophilic
	Passenger Domain	Asp165Gly	Polar to nonpolar
	Serum Resistance Domain	180–185 NTHNINK monomer to NTHNINK dimer	Unknown

^1^ NS, no structural or functional change occurs as a result of this change.

## Discussion

In this study, we describe genotyping of cutaneous leg ulcer *H*. *ducreyi* strains using an MLSA approach that provides information for the genetic basis of the population structure (Table 2). Although the data set is limited to four CLU strains (most of the prior work done on this syndrome was solely PCR-based, limiting the number of available isolates), it does shed light on the relationship of these strains to genital ulcer disease (GUD) strains of *H*. *ducreyi*. The 11 loci around the *H*. *ducreyi* chromosome fall into two groups, *16s rDNA*, *wecA*, *recA*, *ompA2*, *lspA2*, *fgbA*, *dltA*, and *cpxR*, which contain little variation from class I, and *ncaA*, *dsrA*, and *hgbA*, which contain information that differentiates CLU strains from class I *H*. *ducreyi* (Table 4 and Fig. 2).

With the exception of the *dsrA* and *hgbA* loci, all other class I and CLU loci included in the study were either under neutral or purifying selection typically associated with loci in MLSA analysis (Table 3) [19]. The positive selection identified in *dsrA* and *hgbA* is likely due to their association with virulence in humans [24,25]. HgbA is necessary due to its role as a hemoglobin receptor, aiding *H*. *ducreyi* in the acquisition of iron which is required for survival. DsrA is necessary for full virulence in humans due to its roles in serum resistance and fibrinogen binding, helping the bacterium to evade the innate immune response and adhere to the body during pathogenesis. The various amino acid changes within DsrA’s passenger domain responsible for fibrinogen binding will likely influence its ability to bind to fibrinogen, warranting further investigation (Table 5) [23,26].

*H*. *ducreyi* has long been recognized as the etiologic agent of the sexually transmitted infection chancroid, and more recently the causitive agent of chronic, cutaneous limb ulceration afflicting children and adults. *H*. *ducreyi* has been previously described to form two classes based on DNA sequencing and proteomic analyses, yet despite this evolutionary divergence, the classes are associated with the same clinical syndrome. The CLU strains mark the first *H*. *ducreyi* strains isolated whose infection patterns differ from those of GUD class I and class II strains, though the nature of the bacterium as a skin pathogen makes this change plausible.

The presence of unique and informative nucleotide diversity within CLU strains, which form a differentiable subclade from the class I GUD clade, suggests that these isolates may be evolving differently than others (Fig. 2). Using an evolutionary rate of 1% change per million years [22,27], CLU strains diverged from class I approximately 0.355 million years before present, making them the most recently diverged *H*. *ducreyi* strains. Comparatively, class I strains diverged from class II ∼ 5 million years ago, which predates the separation of human and chimpanzee lineages [17,28]. This calculation disproves the notion that the cutaneous strains are older clones of *H*. *ducreyi* [29]. Instead this may be due to specific niche or environmental adaptation.

We do not yet know why the CLU strains are closely related phylogenetically to class I strains. There are distinct genotypic and phenotypic differences between class I and II strains [16–18]. In the lab, class I isolates grow faster and the colonies are larger than class II isolates. Class II isolates are underrepresented in strain collections, most likely because they are more difficult to culture. It is possible that class II strains may also cause cutaenous infections but we have not isolated any strains yet due to the difficulty in growing class II strains in vitro.

Previously, *H*. *ducreyi* was thought to be exclusively transmitted through micro abrasions that occur during sexual intercourse. However, human challenges for mutant parent comparisons have been conducted for over fifteen years in which volunteers were inoculated on the upper arm [12]. Patient inoculations are delivered via allergy testing lancets as 10^6^ *H*. *ducreyi* cells did not infect intact skin in inoculation experiments [30]. Even when infected during human challenge, no cases of secondary transmission occurred in 2,123 subject-days of infection [12]. Thus, the question of why CLU strains are able to transmit at rates of 3.2 per 100 cutaneous ulcer cases presents itself [11]. Whole genome sequencing of multiple class I, class II, and CLU strains would likely provide more detail on the genetic mechanism involved in the CLU strains’ unique proliferation, which is likely due to the modification or addition of virulence factors.

## Supporting Information

S1 Table GenBank accession numbers of the genes analyzed in this study. (DOCX)
