# Supplementary material for: Molecular Phylogenetic Analysis of Non-Sexually Transmitted Strains of Haemophilus ducreyi
Source: PLoS One. 2015 Mar 16;10(3):e0118613. doi: 10.1371/journal.pone.0118613 (PMC4361675; doi:10.1371/journal.pone.0118613)
Supplement: S1 Table — (DOCX) [file pone.0118613.s001.docx]

**S1 Table.**  GenBank^1^ accession numbers of the genes analyzed in this study.

| Strain | *16S rDNA* | *cpxR* | *wecA* | *recA* | *dsrA* | *ncaA* | *ompA2* | *lspA2* | *fgbA* | *hgbA* | *dltA* |
| --- | --- | --- | --- | --- | --- | --- | --- | --- | --- | --- | --- |
| NZS1 | KM592077 | KM592091 | KP295201 | KP295207 | KP067782 | KP295212 | KP295209 | KP295197 | KP067778 | KP295195 | KP067774 |
| NZS2 | KM592078 | KM592089 | KP295200 | KP295205 | KP067783 | KP295213 | KP295210 | KP295199 | KP067779 | KP295194 | KP067775 |
| NZS3 | KM592079 | KM592090 | KP295202 | KP295206 | KP067784 | KP295214 | KP295211 | KP295198 | KP067780 | KP295192 | KP067776 |
| NZS4 | KM592080 | KM592084 | KP295203 | KP295204 | KP067785 | KP295215 | KP295208 | KP295196 | KP067781 | KP295193 | KP067777 |
| SSMC57 | X^2^ | X | X | X | X | X | X | X | X | AY606116.2 | AY371544.1 |
| 82-029362 | X | KM592081 | X | X | X | JF806381.1 | X | X | HQ630260.1 | X | X |
| HMC56 | X | X | X | X | AY606125.1 | X | X | X | X | X | AY371542.1 |
| HMC60 | X | X | X | X | AY606126.1 | X | X | X | X | AY606117.2 | X |
| HD188 | X | KM592087 | JF06412.1 | JF806403.1 | X | JF806386.1 | X | JF806370.1 | HQ630259.1 | X | X |
| HMC46 | X | X | X | X | AY606124.1 | X | X | X | X | AY606118.2 | X |
| HMC50 | X | X | X | X | X | X | X | X | X | X | AY371541.1 |
| 6644 | X | KM592082 | JF806405.1 | JF806396.1 | X | JF806382.1 | X | JF806363.1 | HQ630261.1 | X | X |
| C111 | X | X | JF806407.1 | JF806398.1 | X | JF806383.1 | X | JF806365.1 | X | X | X |
| KC57 | M75084.1 | X | X | X | X | X | X | X | X | X | X |
| HMC62 | X | X | X | X | X | X | X | X | X | X | AY371543.1 |
| 85-023233 | X | X | JF806410.1 | JF806401.1 | X | JF806384.1 | X | JF806368.1 | HQ630259.1 | X | X |
| HD183 | X | KM592086 | JF806411.1 | JF806402.1 | X | JF806385.1 | X | JF806369.1 | HQ630258.1 | X | X |
| 35000HP | AE017143.1 | | | | | | | | | | |
| DMC111 | X | KM592085 | X | X | AY612646.1 | AY612645.1 | X | X | X | AY603046.2 | AY371547.1 |
| DMC64 | X | X | JF806409.1 | X | AY606121.1 | AY612646.1 | X | JF806367.1 | HQ630263.1 | AY606115.2 | AY371546.1 |
| SSMC71 | X | X | X | X | AY606120.1 | AY606128.1 | X | X | X | AY603048.2 | AY371548.1 |
| CIP542 | M75078.1 | X | JF806408.1 | JF806399.1 | AF187007.1 | AY606129.1 | X | JF806366.1 | HQ630262.1 | AY603049.2 | AY371545.1 |
| 33921 | AY513483.1 | X | JF806406.1 | JF806397.1 | AY606120.1 | AY612647.1 | X | JF806364.1 | HQ630264.1 | X | X |
| HMC112 | X | KM592088 | JF806413.1 | JF806404.1 | AY606122.1 | X | X | JF806371.1 | HQ630265.1 | AY60304.2 | X |
| CIPA75 | X | X | X | JF806400.1 | AF187002.1 | X | X | X | X | X | X |
| CIPA77 | X | X | X | X | AF187003.1 | X | X | X | X | X | X |
| V1157 | X | X | X | X | AF187009.1 | X | X | X | X | X | X |
| M90-02 | X | X | X | X | AF187008.1 | X | X | X | X | X | X |

^1^ http://www.ncbi.nlm.nih.gov/genbank/

^2^ X, gene not used in this study
